# Trends in forensic microbiology: From classical methods to deep learning

**DOI:** 10.3389/fmicb.2023.1163741

**Published:** 2023-03-30

**Authors:** Huiya Yuan, Ziwei Wang, Zhi Wang, Fuyuan Zhang, Dawei Guan, Rui Zhao

**Affiliations:** ^1^Department of Forensic Analytical Toxicology, China Medical University School of Forensic Medicine, Shenyang, China; ^2^Liaoning Province Key Laboratory of Forensic Bio-Evidence Science, Shenyang, China; ^3^Department of Forensic Pathology, China Medical University School of Forensic Medicine, Shenyang, China

**Keywords:** forensic microbiology, forensic medicine, machine learning, deep learning, artificial intelligence

## Abstract

Forensic microbiology has been widely used in the diagnosis of causes and manner of death, identification of individuals, detection of crime locations, and estimation of postmortem interval. However, the traditional method, microbial culture, has low efficiency, high consumption, and a low degree of quantitative analysis. With the development of high-throughput sequencing technology, advanced bioinformatics, and fast-evolving artificial intelligence, numerous machine learning models, such as RF, SVM, ANN, DNN, regression, PLS, ANOSIM, and ANOVA, have been established with the advancement of the microbiome and metagenomic studies. Recently, deep learning models, including the convolutional neural network (CNN) model and CNN-derived models, improve the accuracy of forensic prognosis using object detection techniques in microorganism image analysis. This review summarizes the application and development of forensic microbiology, as well as the research progress of machine learning (ML) and deep learning (DL) based on microbial genome sequencing and microbial images, and provided a future outlook on forensic microbiology.

## Introduction

Microorganisms, including viruses, bacteria, and fungi, are ubiquitously distributed and form diverse and rich communities (Oliveira and Amorim, 2018). Forensic microbiology is a discipline that deals with the study of microbiology in the context of forensic investigation (Kuiper, 2016). Its applications mainly focus on the diagnosis of cause of death, inference of crime location, estimation of postmortem interval (PMI), and individual identification.

Numerous studies have explored the application of microorganisms in forensic practice. Certain specific microorganisms have been proven to contribute to the determination of various causes of death, including drowning (Lucci et al., 2008; Kakizaki et al., 2010; Tie et al., 2010; Huys et al., 2012; Lee et al., 2016, 2017; Rácz et al., 2016; Wang et al., 2020), poisoning (Butzbach, 2010; Skopp, 2010; Grad et al., 2012; Han et al., 2012; Butzbach et al., 2013; Sastre et al., 2017), and hospital-acquired infections (Klevens et al., 2007; Sodhi et al., 2013; Lax et al., 2015; Khan et al., 2017). Considering the distinctive microbial community in soil obtained from a crime scene and other intermediary sites, soil microbiome provides evidence for inferring the geolocation (Meyers and Foran, 2008; Costello et al., 2009; Jesmok et al., 2016; Habtom et al., 2019; Yang et al., 2021). Moreover, since microorganisms contribute to postmortem decomposition and have a succession that follows a predictable pattern (Hauther et al., 2015; Javan et al., 2016; DeBruyn and Hauther, 2017; Lutz et al., 2020; Scott et al., 2020), forensic researchers began to explore the feasibility of microbiome succession for inferring the postmortem interval over the past few years. Some studies used machine learning and other technologies to establish models for PMI estimation based on microorganisms, which actually improve the accuracy of PMI inference (Pechal et al., 2014; Johnson et al., 2016; Liu et al., 2020; Zou et al., 2020). In addition, the microbiome composition in humans varies in body location (Kong and Segre, 2012); host characteristics, such as sex, age, and lifestyle (Ross et al., 2017); and skin care status (Bouslimani et al., 2019). Therefore, individuals may be identified using skin, hair, and body fluid microbiomics (Lax et al., 2014; Bäckhed et al., 2015; Wu et al., 2016; Schmedes et al., 2017; Willis et al., 2018).

In this review, we aim to summarize the developmental progress of forensic microbiology from classical methods to high-throughput data combined with artificial intelligence technologies and discuss the outlook for the future.

## Development of forensic microbiology

Forensic microbiology first gained global recognition in 2001 as a result of the *Bacillus anthracis* attacks through the USA postal service. In previous studies on forensic microbiology, forensic microbiological technologies were not specifically described, except for agar cultures for bacteria and fungi combined with PCR for certain microorganisms (Aoyagi et al., 2009; Huys et al., 2012; Uchiyama et al., 2012; Tuomisto et al., 2013; Can et al., 2014; Hauther et al., 2015; Yu et al., 2021a,b). To date, though more than 2,460 different species are presented in The Ribosomal Database Project stores (Maidak et al., 2000), most microbes in the environment have not been described and accessed for biotechnology. Few viable bacteria can be cultivated on artificial media (Kimura and Nobutada, 2006; Cecchini et al., 2012). Furthermore, traditional microbial culture highly relies on culture conditions and has limitations in the analysis of microbial community composition.

The 16/18S rRNA is the common marker for microbial classification and identification. Fluorescence *in situ* hybridization (FISH) (Langendijk, 1995) and denaturing gradient gel electrophoresis (DGGE) (Muyzer et al., 1993) are used to detect the specific 16S rRNA and gain access to unculturable microbes. Sanger sequencing technology (Sanger et al., 1977), also known as first-generation sequencing technology, allows for the wide use of 16S rRNA gene sequencing in bacterial taxonomy and leads to the discovery of a large number of new microbial taxa. Next-generation sequencing technology (NGS) can measure tens of thousands to millions of DNA simultaneously and can provide a high-throughput microbiome database (Metzker, 2010). With the development of sequencing technology, third-generation sequencing technology has more advantages in the study of community diversity due to its ultra-long sequencing reading (Franzén et al., 2015). However, the 16S rRNA test results are assembled into operational taxonomic units (OTUs). Most high-throughput sequencing microbiome data could not be identified and classified due to the limitation of the referenced genomes and genetic datasets (Yooseph et al., 2013; Afshinnekoo et al., 2015). Using targeted 16S rRNA with short amplifiers could not achieve reliable resolution at the species level, and full-length 16S rRNA sequences do not necessarily reduce this limitation (Forney et al., 2004). In addition, whole-genome shotgun (WGS) targets all gene content in microbial ecosystems and can differentiate microbial species and taxa to a greater extent than 16S rRNA amplicons (Schloissnig et al., 2013; Franzosa et al., 2015).

**Figure 1:**
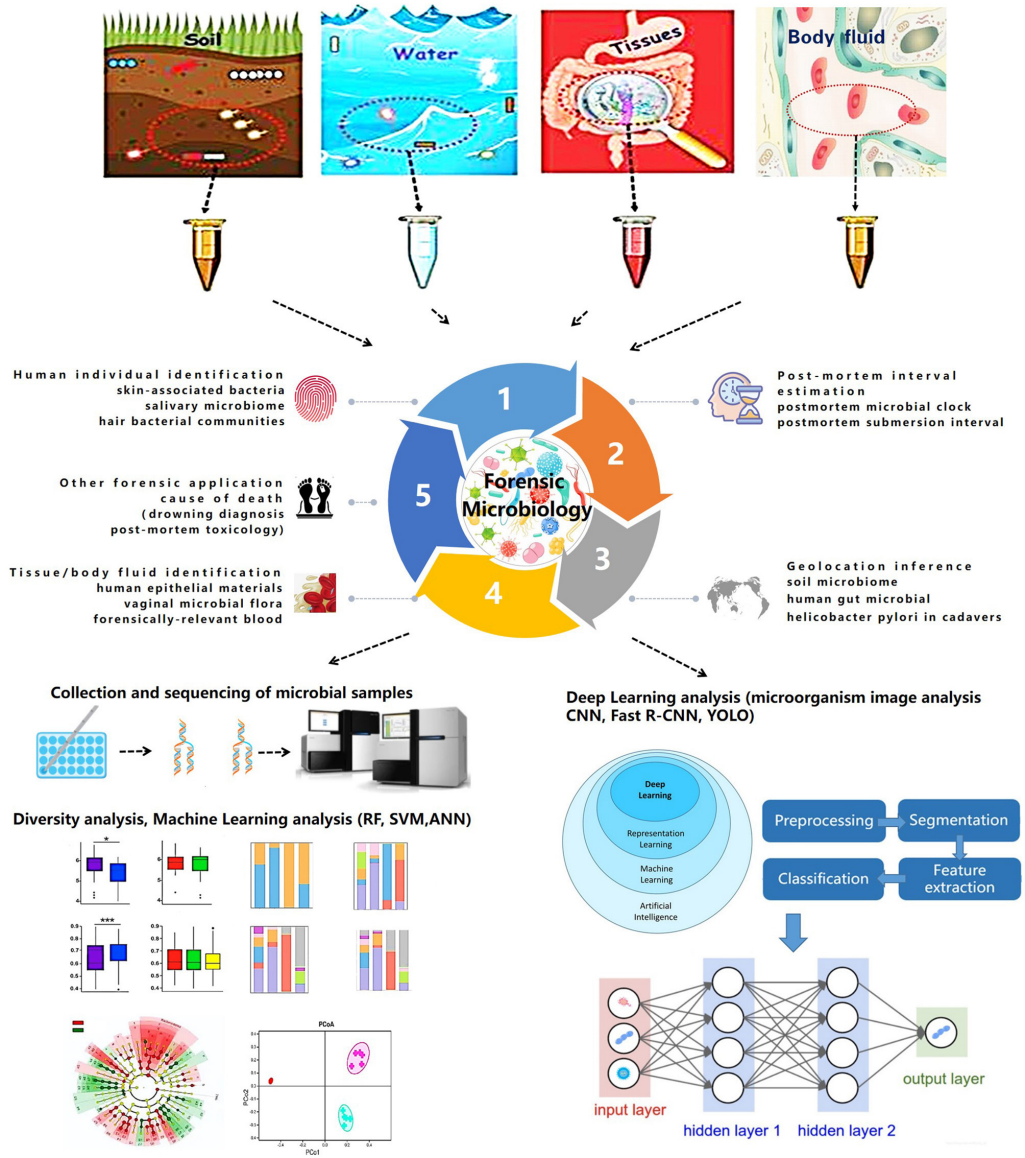
Trends in forensic microbiology.

Though the expanding role of NGS and WGS combined with artificial intelligence will likely be a routine tool in forensic microbiology, the isolation, detection, and confirmation of specific microbes (pathogens or colonies) and the use of nucleic acid sequencing remain less relevant in resolving forensic challenges. Some investigators suggest the use of nanotechnology to design biosensors for the identification of foreign pathogens. Recently, microbial computer image analysis technology combined with machine learning and deep learning elucidated the identification of specific microbiomes (Ma et al., 2022). Moreover, some challenges related to standardization, validation, and procedural and bioinformatic pipelines persist in the study of forensic microbiology (Roesch et al., 2009; Lauber et al., 2010; Wu et al., 2010). Further advancements in technology will continue to improve the application of microbiology in forensic medicine. The trend in forensic microbiology is summarized in Figure 1.

## Applications of machine learning in forensic microbiology

Establishing a prognostic model and predicting the dynamic succession of microbial communities have improved research in forensic microbiology. High-throughput microbiotic datasets combined with machine learning extend the application of microbiomes in forensic issues. Traditional statistical methods could only determine the general composition of a microbial community and its basic succession, whereas machine learning models achieve quantitative analysis and accurate prediction (Zou et al., 2020). Currently, different machine learning models, including random forest (RF), support vector machine (SVM), and AdaBoost, are used in the field of forensic microbiology, and they have become a promising strategy for several forensic events.

One main use of forensic microbiology is human individual identification (Fierer et al., 2010; Lax et al., 2015; Leake et al., 2016; Williams and Gibson, 2017; Yang et al., 2019). In cases without blood or tissue evidence, a microbiological approach provides a breakdown of situations with the detection of the microbiome on skin, saliva, hair, or objects that have been touched. UniFrac metric (Fierer et al., 2010) and hierarchical clustering (Leake et al., 2016) are used in determining skin-associated bacteria and salivary microbiomes. In an RF model used for investigating pubic hair bacteria, the accuracy of individual identification was 2.7 ± 5.8%, and the gender accuracy was 1.7 ± 5.2% (Williams and Gibson, 2017).

In addition, machine learning can be used for PMI estimation in microbial forensics (Johnson et al., 2016; Liu et al., 2020, 2021; Cao et al., 2021; Cartozzo et al., 2021; Randall et al., 2021; Zhang et al., 2021; Kaszubinski et al., 2022; Zhao et al., 2022). RF models are widely used for PMI estimation and postmortem submersion interval (PMSI) diagnosis (Metcalf et al., 2013; Pechal et al., 2014; Cartozzo et al., 2021; Randall et al., 2021; Zhang et al., 2021; Kaszubinski et al., 2022; Zhao et al., 2022). In 2013, Metcalf et al. (2013) first proposed the concept of a “microbial clock” to infer PMI by setting up regression models using an RF. In addition, artificial neural network (ANN) models are used to infer PMI based on the microbiotic OTU data. In 2020, Liu et al. combined microbial community characterization and machine learning algorithms (RF, SVM, and ANN) to investigate microbial succession patterns during corpse decomposition and estimate PMI with a mean absolute error (MAE) of 1.5 ± 0.8 h within 24 h.

Available evidence demonstrates that forensic microbiology with machine learning can be used to infer geolocation to a certain extent (McNulty et al., 2004; Kersulyte et al., 2010; Blaser et al., 2013; Nagasawa et al., 2013; Tyakht et al., 2013; Escobar et al., 2014; Li et al., 2014; Suzuki and Worobey, 2014; Huang et al., 2020). Human gut microbiota plays a significant role in geolocation inference, which is supported by correlations based on traditional statistical methods, such as Wilcoxon's rank-sum test (Suzuki and Worobey, 2014) and analysis of similarities (ANOSIM) (Escobar et al., 2014). In 2014, UniFrac, network, and ANOSIM were used to analyze the human saliva microbiome. The variance between individuals was 6.75–10.21% (Li et al., 2014). In 2020, Huang et al. (2020) applied a machine learning framework to determine geolocations with an accuracy of 86%.

As previously stated, machine learning can be used to establish models for tissue/body fluid identification (Costello et al., 2009; Benschop et al., 2012; Lopez et al., 2019, 2020; Salzmann et al., 2021). Lopez et al. (2019) used ANN to identify different human epithelial biomaterials with AUC values of 0.99, 0.99, and 1 for skin, oral, and vaginal secretions, respectively. Later in 2020, deep neural network (DNN) was additionally used to identify human forensically relevant blood samples successfully (Lopez et al., 2020).

Investigative forensic microbiology with machine learning has practical applications in other aspects. Concerns related to drowning diagnosis (Huys et al., 2012; Wang et al., 2020), postmortem toxicology (Kaszubinski et al., 2020), and disease diagnosis are summarized in Table 1. Although the expanding effect of machine learning on NGS and WGS databases is without much doubt, some models derived from different machine learning models still mean less to the actual situation, which limits their application in forensic practice. The current challenges of machine learning are insufficient training samples and an imperfect microbial databank.

**Table 1:** Applications of artificial intelligence in forensic microbiology.

**Forensic issue**	**Animal model**	**Sampling location**	**AI model**	**Model performance**	**References**
**Human individual identification**					
	Human	Skin, Keyboard, Smartphone screen	RF	Accuracy around 90%	Yang et al., 2019
	Human	Shoes and phones	RF	Error ratio 3.6	Lax et al., 2015
	Human	Skin, computer keyboards	ANOSIM	Unweighted PC_O_1 (17%) PC_O_2 (6.5%)	Fierer et al., 2010
				Weighted PC_O_1 (61%) PC_O_2 (19%)	
	Human	Saliva	Hierarchical clustering		Leake et al., 2016
	Human	Hair	RF	Individual 2.7% ± 5.8%	Williams and Gibson, 2017
				Gender 1.7% ± 5.2%	
**Post-mortem interval estimation**					
	Mice	Cecum	RF	MAE 20.01 h	Liu et al., 2021
	Mice	Brain, heart, and cecum	RF, SVM, ANN	MAE within 24 h	Liu et al., 2020
	Swine	Bone	RF	Variation >80%	Kaszubinski et al., 2022
	Pig	Bone	RF	RMSE ± 104 days Rib	Randall et al., 2021
				RMSE ± 63 days Scapulae	
	Pig	Bone	RF	RMSE ± 27 days Rib	Cartozzo et al., 2021
				RMSE ± 29 days Scapulae	
	Rat	Oral	RF	*R*^2^ = 93.94%	Zhao et al., 2022
	Rat	Gravesoil, rectum and skin	RF	MAE 1.82 days in Gravesoil	Zhang et al., 2021
				MAE 2.06 days in Rectum	
	Human	Nasal cavity and ear canal	K-neighbors regression (KNR), Ridge regression (RR), Lasso regression (LR), Elastic net regression (ENR), Random forest regression (RFR), Bayesian ridge regression (BRR)	MAE ± 55ADD	Johnson et al., 2016
	Rat	Cecum	Partial least squares (PLS)	RMSE within 9 days	Cao et al., 2021
**Geolocation inference**					
	Human	Gut	Spearman's correlations	Positive correlation between Firmicutes abundance and latitude (ρ = 0.857, *p* < 0.0001)	Suzuki and Worobey, 2014
			Wilcoxon rank sum test	Negative correlation between Bacteroidetes and latitude (ρ = −0.637, *p* = 0.001)	
	Human	Gut	ANOSIM	ADONIS: *R*^2^ = 0.22, *P* = 0.001	Escobar et al., 2014
				ANOSIM: *R* = 0.78, *P* = 0.001	
	Human	Gut	ANOSIM	Russian and the US, Danish and Chinese groups *R* = 0.74, 0.50 and 0.26, respectively *P* = 9.999 * 10^−5^	Tyakht et al., 2013
	Human	Skin	ANOVA, ANOSIM, PERMANOVA	US vs. VZ; ANOSIM *P* < 0.001; PERMANOVA *P* < 0.001 for both unweighted and weighted measures	Blaser et al., 2013
	Human	Saliva	UniFrac, network, ANOSIM	Variance between individuals: 6.75–10.21% within individuals: 89.79–93.25%	Li et al., 2014
				ANOSIM statistic: *R* = −0.0935, *P*-value = 0.7386	
	Human	Subways and urban biomes	Logistic regression model with L_2_ regularization	Accuracy 86%	Huang et al., 2020
**Tissue/body fluid identification**					
	Human	Skin, oral and vaginal	ANN	AUC values of 0.99 for skin, 0.99 for oral, and 1 for vaginal secretion	Lopez et al., 2019
	Human	Blood	DNN	0.978 for nasal blood	Lopez et al., 2020
				0.978 for finger-prick blood	
	Human	Vagina	Microarray analysis	121 of the 389 probes detected	Benschop et al., 2012
	Human	Blood, menstrual blood, saliva, semen, and vaginal secretion	Lasso regression analysis	26 taxa showed high predictive value for TsD	Salzmann et al., 2021
**Other forensic application**					
**Drowning diagnosis**					
	Rat	Skin, cardiac blood, lung, and liver	Unweighted UniFrac-based PCoA	PCo1 60.27% PCo2 19.15% (skin) PCo1 52.66% PCo2 15.98% (lung) PCo1 50.52% PCo2 18.37% (blood)	Wang et al., 2020
**Post-mortem toxicology**					
	Human	Nose, mouth, rectum, ears, eyes	Beta-dispersion	Cardiovascular disease and drug-related deaths correctly classified in 79%	Kaszubinski et al., 2020
**Microorganism image analysis**					
	Fungus		CNN	Accuracy 94.8%	Tahir et al., 2018
	Bacteria		CNN	Accuracy 96%	Treebupachatsakul and Poomrittigul, 2019
	Actino		CNN, ResNet	Accuracy 80.8% to 80.1%	Sajedi et al., 2019
	Diatoms		R-CNN, YOLO	F-measure of YOLO 84%	Bueno et al., 2018
	Diatoms		Inception V3	Identification rate 89.6%	Zhou et al., 2019
	Diatoms		YOLO, SegNet	Specificity, sensitivity, precision	Salido et al., 2020
	Diatoms		SegNet, Mask R-CNN	Precision, sensitivity, specificity	Ruis-Santaquiteria et al., 2020
	Diatoms		CNN	Validation set accuracy 97.33%	Zhou et al., 2019
	Cyanobacteria		Fast R-CNN	Average precision 0.929	Baek et al., 2020
					*R*^2^ value of 0.775
	Soybean cyst nematode eggs		CNN	Average accuracy 94.33%	Akintayo et al., 2016
	Tuberculosis		CNN	Precision 78.4%	Oomman et al., 2018
	Cell in blood		Faster R-CNN	Total accuracy 98%	Hung et al., 2017

## Deep learning for microorganisms

Compared with machine learning, deep learning can realize automatic feature learning through advanced network structure by combining several simple modules (Lecun et al., 2015; Ma et al., 2022). The deep learning process generally needs a large amount of training data followed by the formation of suitable neural networks. Previous studies have proven that convolutional neural networks (CNNs) and their derivative models allow for accurate tissue-type classification and microbiome identification, which could further extend the diagnosis of microbially caused death and personal identification in forensic science. A recent study constructed a CNN model through micorbiome analysis to classify three different human epithelial materials of skin, oral, and vaginal origins (Lopez et al., 2019). Other researchers proposed and designed the CNN based on a system for detecting fungi, parasite ova, and bacteria (Akintayo et al., 2016; Tahir et al., 2018). The accuracy for identifying fungi, soybean cyst nematode eggs, and bacteria was 94.8% (Tahir et al., 2018), 94.33% (Akintayo et al., 2016), and 96% (Treebupachatsakul and Poomrittigul, 2019), respectively.

Recent studies have demonstrated that CNN models could differentiate bacteria and algae based on microbiome images. The application of microbial computer image analysis mainly focuses on the segmentation, clustering, classification, and counting of microorganisms (Ma et al., 2022). The CNN models based on images for microbiome identification are supported by the study of Panicker (Oomman et al., 2018), which could detect tuberculosis in microscopic sputum smear images with a precision of 78.4%. In addition, deep learning on image analysis is mainly focused on the identification of diatoms and algae, which would provide a potential strategy for the diagnosis of drowning. In 2018, Bueno et al. (2018) compared the functions of R-CNN and you only look once (YOLO) in diatom detection; the F-measure of YOLO was 84%. In 2019, Huang et al. applied and trained a CNN model based on the GoogLeNet Inception V3 architecture to identify diatoms with a validation rate of 97.33% (Zhou et al., 2019, 2020), which indicates that DL is an efficient and low-cost automated diatom detection technology. In 2020, YOLO and SegNet were compared by Salido et al. (2020). In 2020, Ruis-Santaquiteria et al. (2020) found that Mask-RCNN and SegNet models are capable of segment diatoms from the same raw images used for manual identification, without any cropping or preprocessing step. With the assistance of necessary operation systems, such as the Center for Microbial Ecology Image Analysis System (Dazzo and Niccum, 2015), image analysis of microorganisms would contribute more to forensic microbiology due to its convenience and operational speed (Ma et al., 2022).

## Future outlook

Forensic microbiology is still in its infancy. Advanced technologies, such as NGS and WGS, have provided a sufficient dataset that could not be imagined previously. Further advancements in technology will continue to improve the capacity for forensic microbial investigations. In addition, artificial intelligence has promoted the technological innovation of forensic microbiology in the past decades. With the accumulation of genomic sequencing datasets and microbial images, deep learning will exert greater power in achieving better prognostic models with higher accuracy. However, there are several issues that deserve further discussion.

1. Advantages and defects of AI

Based on the current microbiota database, ML and DL have the quick and automatic capability of relatively accurate prediction compared with the conditional methods. The “black box” of AI methods would not completely and theoretically characterize the real results. Although the new algorithms of AI will shed light on microbiotic analysis for forensic purposes, the basic strategy is focused on the bigger microbiota database to promote AI to work better.

2. Key points for establishing the forensic microbiota database

Considering the variation of microbial communities, the development of a forensic microbial bank is urgently needed. The sampling and sequencing procedures of forensic microorganisms still need to be further standardized. In addition, due to the complex influence of sample types, locations, environmental factors, and postmortem changes, combining the microbiome data from the experimental animals, human samples, and certain materials from the crime scene should be considered. Investigators should carry out multi-center cooperation.

3. Microbiome combined with other methods to solve forensic problems

The selection of differential microbes by bioinformatic technologies could better disclose microbial markers. AI could establish models for predicting forensic issues. Based on the specific microbes that could characterize the different body fluids and environments, even individual identification, targeted PCR testing of the selected microorganisms could be explored to improve the efficiency and accuracy of forensic problems in future.

## Author contributions

RZ and DG designed the manuscript and edited the manuscript. HY and ZiW wrote the manuscript. ZhW and FZ searched, edited, and reviewed the literature. All authors have read and commented on the manuscript. All authors contributed to the article and approved the submitted version.
